# Supplementary material for: Deep Visual Proteomics maps proteotoxicity in a genetic liver disease
Source: Nature. 2025 Apr 16;642(8067):484–91. doi: 10.1038/s41586-025-08885-4 (PMC12158776; doi:10.1038/s41586-025-08885-4)
Supplement: Supplementary file 2 — Reporting Summary [file 41586_2025_8885_MOESM2_ESM.pdf]

nature portfolio

Corresponding author(s): FAR, MM

Last updated by author(s): Mar 30, 2025

Reporting Summary

Nature Portfolio wishes to improve the reproducibility of the work that we publish. This form provides structure for consistency and transparency in reporting. For further information on Nature Portfolio policies, see our Editorial Policies and the Editorial Policy Checklist.

Statistics

For all statistical analyses, confirm that the following items are present in the figure legend, table legend, main text, or Methods section.

n/a	Confirmed	
☐	☒	The exact sample size (n) for each experimental group/condition, given as a discrete number and unit of measurement
☐	☒	A statement on whether measurements were taken from distinct samples or whether the same sample was measured repeatedly
☐	☒	The statistical test(s) used AND whether they are one- or two-sided<br>*Only common tests should be described solely by name; describe more complex techniques in the Methods section.*
☐	☒	A description of all covariates tested
☐	☒	A description of any assumptions or corrections, such as tests of normality and adjustment for multiple comparisons
☐	☒	A full description of the statistical parameters including central tendency (e.g. means) or other basic estimates (e.g. regression coefficient) AND variation (e.g. standard deviation) or associated estimates of uncertainty (e.g. confidence intervals)
☐	☒	For null hypothesis testing, the test statistic (e.g. F, t, r) with confidence intervals, effect sizes, degrees of freedom and P value noted<br>*Give P values as exact values whenever suitable.*
☐	☒	For Bayesian analysis, information on the choice of priors and Markov chain Monte Carlo settings
☐	☒	For hierarchical and complex designs, identification of the appropriate level for tests and full reporting of outcomes
☐	☒	Estimates of effect sizes (e.g. Cohen's d, Pearson's r), indicating how they were calculated

Our web collection on statistics for biologists contains articles on many of the points above.

Software and code

Policy information about availability of computer code

Data collection	Zeiss ZEN Blue (3.7.97.07000), PerkinElmer Harmony v4.9, Single-Cell Technologies BIAS (September 2023), Leica LMD Beta10, Orbitrap Astral Tune Application 1.0.100.40, Cellpose 2.0, scPortrait, ConvNext, huggingface v4.26
Data analysis	DIA-NN v1.8.1, , directLFQ v0.2.19, R v4.4.1, limma v3.60.3, PCAtools v2.16.0, WebGestalt 2024, umap-learn 0.5.6, scikit-learn 1.4.2, pandas 2.2.1, numpy 1.26.4

For manuscripts utilizing custom algorithms or software that are central to the research but not yet described in published literature, software must be made available to editors and reviewers. We strongly encourage code deposition in a community repository (e.g. GitHub). See the Nature Portfolio guidelines for submitting code & software for further information.

Data

Policy information about availability of data

All manuscripts must include a data availability statement. This statement should provide the following information, where applicable:

- Accession codes, unique identifiers, or web links for publicly available datasets
- A description of any restrictions on data availability
- For clinical datasets or third party data, please ensure that the statement adheres to our policy

The mass spectrometry proteomics data have been deposited to the ProteomeXchange Consortium via the PRIDE partner repository with the dataset identifier PXD054440. The R and Python code used in this study is documented at https://github.com/MannLabs/Proteotoxicity. Imaging data of explant and morphological clusters has been deposited to BioStudies with the identifier S-BIAD1523.

Research involving human participants, their data, or biological material

Policy information about studies with human participants or human data. See also policy information about sex, gender (identity/presentation), and sexual orientation and race, ethnicity and racism.

Reporting on sex and gender	Clinical metadata including sex are summarised in Extended Data Fig. S1A. Both male and female samples were included without prior selection due to the small cohort size.
Reporting on race, ethnicity, or other socially relevant groupings	Socially constructed categorization was not taken into consideration. Patient material was sampled in Odense, Denmark, and Aachen, Germany, and represent the patient cohort in the larger region.
Population characteristics	Clinical metadata including age, alcohol consumption, diabetes and BMI are summarised in Extended Data Fig. S1A. Individual samples were explicitly de-identified to to comply with European and country-specific General Data Protection Regulation (GDPR).
Recruitment	Odense patient recruitment – Patients were recruited through the Danish patient organization (Alfa-1 Denmark) and clinical departments for liver and lung diseases as part of a cohort study. The cohort was designed to investigate liver health among non-pregnant adults (minimum age 18 years) diagnosed with AATD of any genotype and carrier status. This specific study includes 16 individuals diagnosed with Pi*ZZ who consented to undergo the procedure. Participants without a history of liver transplant or decompensated cirrhosis were offered a percutaneous liver biopsy. The patients underwent liver core needle biopsies at Odense University Hospital (OUH) between 2017 and 2021.<br>Aachen patient recruitment – The recruitment of patients is described in detail in reference 34. Of this cohort, the present study includes 19 individuals diagnosed with Pi*ZZ, of whom 14 underwent liver core needle biopsies due to medical indication and five received a liver transplantation due to end-stage liver disease.
Ethics oversight	Odense: The study was approved by the Danish Ethical Committee (S-20160187), and participants gave informed consent prior to enrollment. Aachen: thical approval was provided by the institutional review board of Aachen University (EK 173/15). All participants provided written informed consent and were treated following the ethical guidelines of the Helsinki Declaration (Hong Kong Amendment) as well as Good Clinical Practice (European guidelines).

Note that full information on the approval of the study protocol must also be provided in the manuscript.

Field-specific reporting

Please select the one below that is the best fit for your research. If you are not sure, read the appropriate sections before making your selection.

☒ Life sciences ☐ Behavioural & social sciences ☐ Ecological, evolutionary & environmental sciences

For a reference copy of the document with all sections, see nature.com/documents/nr-reporting-summary-flat.pdf

Life sciences study design

All studies must disclose on these points even when the disclosure is negative.

Sample size	Thirty-four of 35 available patient Pi*ZZ samples were included. One sample was excluded due to its outlier position on a PCA.
Data exclusions	MS samples were included if the number of protein groups exceeded (a) the mean minus 1.5 standard deviations for DVP, resulting in 5.9% (6/102) dropouts; (b) the mean minus 0.5 standard deviations for DVP-ML samples; (c) a fitted logarithmic curve minus 1.5 interquartile ranges for scDVP, taking the relation between size and proteomic depth into account, resulting in 15.4% (40/259) dropouts. The lower cutoffs were selected after manual inspection of the data distribution. For morphology-guided DVP, one explant section was removed from analysis due to too few AAT-positive cells in the section.
Replication	Only one technical replicate was used and analysed per patient biopsy (see Supplementary Table S1).

Randomization	Patient samples were processed in random order. MS acquisition was not randomized and MS performance was tightly monitored with HeLa QC runs before, during, and after each experimental block.
Blinding	Cell selection was automated and driven by k means clustering or convolutional neural networks without human input. Investigators were blinded for laser microdissection. Data analysis was not blinded to ensure correct handling of biological positive controls.

Reporting for specific materials, systems and methods

We require information from authors about some types of materials, experimental systems and methods used in many studies. Here, indicate whether each material, system or method listed is relevant to your study. If you are not sure if a list item applies to your research, read the appropriate section before selecting a response.

Materials & experimental systems

n/a	Involved in the study
☐	☒ Antibodies
☒	☐ Eukaryotic cell lines
☒	☐ Palaeontology and archaeology
☒	☐ Animals and other organisms
☐	☒ Clinical data
☒	☐ Dual use research of concern
☒	☐ Plants

Methods

n/a	Involved in the study
☒	☐ ChIP-seq
☒	☐ Flow cytometry
☒	☐ MRI-based neuroimaging

Antibodies

Antibodies used	PRIMARY: Mouse IgG1 monoclonal AAT 2C1 (1:200, Hycult HM2289); Rabbit recombinant anti-pan cadherin [EPR1792Y] (1:200, Abcam ab51034)<br>SECONDARY: Goat anti-mouse IgG1 (1:400 Invitrogen A21127); Goat anti-rabbit AF647 (1:400, Invitrogen A21245)
Validation	The mouse anti-human AAT monoclonal antibody 2C1 was tested positive in an ELISA assay according to the manufacturer: https://www.hycultbiotech.com/product/alpha-1-antitrypsin-human-mab-2cl/. The rabbit monoclonal anti-pan cadherin antibody [EPR1792Y] was tested by the manufacturer Abcam (ab51034) in Western blot on recombinant protein, by flow cytometry against an isotype control, and through OI-RD scanning.

Clinical data

Policy information about clinical studies

All manuscripts should comply with the ICMJE guidelines for publication of clinical research and a completed CONSORT checklist must be included with all submissions.

Clinical trial registration	N/A
Study protocol	N/A
Data collection	N/A
Outcomes	N/A

Plants

Seed stocks	N/A
Novel plant genotypes	N/A
Authentication	N/A
